# Flavonoid-membrane Interactions: A Protective Role of Flavonoids at the Membrane Surface?

**DOI:** 10.1080/10446670410001722168

**Published:** 2005-03

**Authors:** Patricia I. Oteiza, Alejandra G. Erlejman, Sandra V. Verstraeten, Carl L. Keen, César G. Fraga

**Affiliations:** 1 Department of Biological Chemistry IQUIFIB (UBA-CONICET) School of Pharmacy and Biochemistry University of Buenos Aires Buenos Aires Argentina; 2 Department of Nutrition University of California Davis CA USA; 3 Department of Environmental Toxicology University of California Davis CA USA; 4 Physical Chemistry-PRALIB School of Pharmacy and Biochemistry University of Buenos Aires Buenos Aires Argentina

## Abstract

Flavonoids can exert beneficial health effects through multiple mechanisms. In this paper, we address the important, although not fully understood, capacity of flavonoids to interact with cell membranes. The interactions of polyphenols with bilayers include: (a) the partition of the more non-polar compounds in the hydrophobic interior of the membrane, and (b) the formation of hydrogen bonds between the polar head groups of lipids and the more hydrophilic flavonoids at the membrane interface. The consequences of these interactions are discussed. The induction of changes in membrane physical properties can affect the rates of membrane lipid and protein oxidation. The partition of certain flavonoids in the hydrophobic core can result in a chain breaking antioxidant activity. We suggest that interactions of polyphenols at the surface of bilayers through hydrogen bonding, can act to reduce the access of deleterious molecules (i.e. oxidants), thus protecting the structure and function of membranes.


*Clinical & Developmental Immunology*, March 2005; 12(1): 19–25

Taylor & Francis

Taylor & Francis Group

### Flavonoid-membrane interactions: A protective role of flavonoids at the membrane surface?

PATRICIA I. OTEIZA[1,2,3], ALEJANDRA G. ERLEJMAN[1], SANDRA V. VERSTRAETEN[1], CARL L. KEEN[2], & CÉSAR G. FRAGA[2,4]

[1]*Department of Biological Chemistry, IQUIFIB (UBA-CONICET), School of Pharmacy and Biochemistry, University of Buenos Aires, Buenos Aires, Argentina,* [2]*Department of Nutrition, University of California, Davis, CA, USA,* [3]*Department of Environmental Toxicology, University of California, Davis, CA, USA, and* [4]*Physical Chemistry-PRALIB, School of Pharmacy and Biochemistry, University of Buenos Aires, Buenos Aires, Argentina*

**Abstract**
Flavonoids can exert beneficial health effects through multiple mechanisms. In this paper, we address the important, although not fully understood, capacity of flavonoids to interact with cell membranes. The interactions of polyphenols with bilayers include: (a) the partition of the more non-polar compounds in the hydrophobic interior of the membrane, and (b) the formation of hydrogen bonds between the polar head groups of lipids and the more hydrophilic flavonoids at the membrane interface. The consequences of these interactions are discussed. The induction of changes in membrane physical properties can affect the rates of membrane lipid and protein oxidation. The partition of certain flavonoids in the hydrophobic core can result in a chain breaking antioxidant activity. We suggest that interactions of polyphenols at the surface of bilayers through hydrogen bonding, can act to reduce the access of deleterious molecules (i.e. oxidants), thus protecting the structure and function of membranes.

**Keywords:** *Flavonoids, polyphenols, membranes, antioxidants, biophysics, review*

### Introduction

In the past, membrane lipids were considered merely as constituents of an inert matrix where the proteins were located, being the latter the effectors of all biological processes. Lipids are now recognized as important mediators in a number of metabolic pathways. For example, sphingomyelin is a chemoprotective agent, impeding the growth of colon neoplasias (Exon and South 2003), and phosphatidylcholine has a key role in the immunomodulation of leukocyte-mediated inflammatory response (Tonks et al. 2001). Changes in membrane rheology can affect multiple biological events that occur at the cellular membrane level, including the activity of certain enzymes, the transport of metabolites, signal transduction events, membrane receptors, endo- and exo-cytosis and interactions of membrane components with the cytoskeleton (Kuo et al. 1990, Tomassoni et al. 1999, Niranjan and Krishnakantha, 2000, Hashimoto et al. 2001). These biological functions of membranes could be severely affected when damaged by reactive oxygen species.

Flavonoids are widely distributed in plant foods, and constitute the most common phenolic compounds in plants. Flavonoids have been described to modulate certain immune processes, such as the inhibition of myelin membrane phagocytosis in multiple sclerosis (Hendricks et al. 2003), and the inhibition of $PGE_2$ (Lina et al. 2003) and IgE (Lim 2003) production. Both in biological and in model membranes, the interaction between the flavonoids and the bilayer results in either the binding at the lipid–water interface, or the distribution in the hydrophobic core of the membrane. The different location of these molecules is determined by their chemical properties. Based on our work and that

Correspondence: P.I. Oteiza, Address: Department of Nutrition, University of California, Davis, Davis, CA 95616, USA. Tel: 1 530 754 6074. Fax: 1 530 752 8966. E-mail: poteiza@ucdavis.edu

ISSN 1740-2522 print/ISSN 1740-2530 online © 2005 Taylor & Francis Group Ltd.
DOI: 10.1080/10446670410001722168

of others we suggest that the more hydrophilic flavonoids can interact at the membrane surface and provide protective actions against different deleterious agents. In the case where the potential harmful substance is an oxidant, flavonoid-membrane interaction may be one of the mechanisms involved in the antioxidant action of flavonoids.

### The interactions of flavonoids with membranes

Based on their chemical structure flavonoids are classified as chalcones, dihydrochalcones, aurones, flavones, flavonols, dihydroflavonols, flavanols, flavandiols, anthocyanidins, isoflavonoids, biflavonoids and other highly polymerized structures (Bravo 1998). Differences in the number and distribution of hydroxyl groups, the polymerization degree (Verstraeten et al. 2003), as well as the presence of a methoxy group in the C ring, can influence the type of interactions that occur between different flavonoids and lipid bilayers. Two possible relevant interactions are: (a) the partitioning of the polyphenol in the non-polar core of the membrane, associated with the hydrophobic nature of the flavonoid; and (b) the interaction of the hydrophilic flavonoids and oligomers with the polar headgroups of lipids at the lipid–water interface, mainly associated with the formation of hydrogen bonds.

### *Partitioning of flavonoids in the hydrophobic portion of membranes*

Depending on their chemical structure, flavonoids can partition in the hydrophobic core of membranes. Among different subclasses of flavonoids, their relative hydrophobicity measured as the partition coefficient between water and olive oil, is higher for the flavones and flavanones than for the flavanols (van Dijk et al. 2000). At the same hydroxylation degree, flavones are more hydrophobic than flavanones. The capacity of these compounds to affect transmembrane potential and pH differences is restricted to the more hydrophobic flavones and flavanones, while the flavonols, quercetin and morin, have no effects on these parameters (van Dijk et al. 2000). Ollila et al. (2002) showed an inverse correlation between the capacity of acacetin, apigenin, *n*-propyl gallate, luteolin, quercetin and myricetin to induce membrane permeabilization (release of calcein entrapped in liposomes) and their retention in a phosphatidylcholine coated column. The authors reported that this correlation was related to the relative hydrophobicity of the compounds (presence of the methoxy group, distribution of hydroxyl groups on the B-ring). The capacity of cinnamic acid and *p*-coumaric acid (4 hydroxy-cinnamic acid) to promote liposome permeability was studied by differential scanning calorimetry (Castelli et al. 1999). The more hydrophobic compound, cinnamic acid, affected membrane permeability while its hydroxylated derivative, with a higher polar character, had no effect (Castelli et al. 1999). Enhancing the hydrophobicity of cinnamic acid by decreasing the pH of the media (which causes the protonation of the carboxyl group) enhanced its permeabilizating effect. Similarly, the embedding of quercetin in bilayers depends on the pH of the media. At acidic pH, quercetin is deeply embedded in planar lipid bilayers (Movileanu et al. 2000), while at physiological pH it interacts with the polar head groups at the water–lipid interface (Terao et al. 1994, Movileanu et al. 2000, Pawlikowska-Pawlega et al. 2003). The flavanones, naringenin and rutin, and a series of isoflavones partition in the hydrophobic portion of liposome bilayers, inducing the loss of membrane fluidity (Arora et al. 2000). The use of fluorescent probes that test lipid packing at different depths in the hydrophobic core showed the highest ordering effect of the flavonoids in the deepest region of the membrane (Arora et al. 2000). At pH 7.4, quercetin, hesperetin and naringenin decreased the transition temperature of dipalmitoylphosphatidylcholine liposomes (Saija et al. 1995). This result indicates that at neutral pH, both flavonoids (hesperetin and naringenin) that distribute in the hydrophobic portion of the membrane, and quercetin, which interacts with the surface of the bilayer, can affect the transition temperature of a membrane (Saija et al. 1995).

### *Interaction of flavonoids and related polymers at the lipid–water interface of membranes*

Flavonoids can vary in their number and distribution of hydroxyl groups, key points of interaction between the polyphenols and the membrane surface at the water–lipid interface through the formation of hydrogen bonds. Using a phosphatidylcholine-coated HPLC column, Ollila et al. (2002) showed that the surface interaction (measured as the retention delay) between eight different flavonoids and the phospholipid was positively correlated with the number of hydroxyl groups in the compounds. In addition, Tsuchiya (2001) demonstrated that epicatechin, which is more hydrophobic than its geometrical isomer catechin, has a higher interaction with membrane lipids.

Van Dijk et al. (2000) evaluated the affinity of flavonoids for liposomes, measuring their capacity to quench the fluorescence of a membrane probe. The affinity of the flavanols morin and quercetin for liposomes was markedly higher than that of the flavanones pinocembrin, naringenin, eriodictyol and hesperitin. This difference was attributed to the planar tridimensional structure of flavanols compared to a tilted configuration of flavanones. The glucosylated derivatives at position 7 of naringenin and eriodictyol showed a higher affinity than the respective aglycons, which is proposed to be due to a torsion of the molecule secondary to glucosylation.

Two highly hydroxylated flavonoids, myrecetin and rutin, did not affect the fluorescence polarization of a probe located in the polar region of phosphatidylcholine liposomes (Ratty et al. 1988). This finding indicates that myrecetin and rutin do not penetrate the bilayer even at the most superficial membrane environment, the polar region.

### The influence of membrane physical properties and cell surface interactions on membrane oxidation

It is known that flavonoids can display antioxidant activity in numerous biological systems (Lotito and Fraga 1998, Lotito et al. 2000, Rice-Evans 2001).

The rate of lipid and protein oxidation in membranes can be modulated by changes in certain physical properties of the bilayer. Membrane fluidity and lateral phase separation are crucial determinants in the rate of membrane oxidation.

An increase in lipid oxidation rates at temperatures below membrane transition temperature ($T_m$) has been observed in association with decreased bilayer fluidity (Mowri et al. 1984, Cervato et al. 1988). Arachidonic acid incorporated into 1-palmitoyl-2-arachidonoyl phosphatidylcholine liposomes is more susceptible to oxidation when the host lipid is in gel phase than in liquid crystalline phase (McLean and Hagaman, 1992). Cholesterol has a dual effect; at temperatures below $T_m$, cholesterol reduces lipid oxidation by increasing membrane fluidity. On the contrary, at temperatures above $T_m$, cholesterol rigidifies the bilayer, leading to higher rates of lipid oxidation (McLean and Hagaman, 1992).

Work from our laboratory demonstrated that metals without redox capacity ($Al^{3+}$, $Ga^{3+}$, $Sc^{3+}$, $Y^{3+}$, $In^{3+}$, $La^{3+}$ and $Be^{2+}$) can stimulate lipid and protein oxidation in the presence of iron as an initiator in synthetic and in brain membranes (Oteiza 1994, Verstraeten et al. 1997a,b). Aluminum and related metals facilitate the formation in the membrane of phosphatidylserine-enriched clusters where the mobility of the acyl chain is restricted. As membranes become more rigid, the motion of fatty acids decreases and the probability of lipid radical interactions with other fatty acids increases, facilitating the propagation of lipid oxidation (Verstraeten et al. 1997, Verstraeten and Oteiza 2000). This effect can be further potentiated by the clustering of lipids with highly peroxidizable chains such as brain phosphatidylserine and by the presence of galactolipids (Verstraeten et al. 1998).

On the other hand, the interaction of certain compounds with the polar interface of the bilayer can protect it from the deleterious effects of pro-oxidants. We recently demonstrated that zinc can protect liposomes from aluminum- and iron-stimulated lipid oxidation (Zago et al. 2000, Zago and Oteiza 2001). This effect can be attributed to a direct binding of zinc to phospholipids which prevents the binding of iron (Zago and Oteiza 2001) and aluminum (Zago et al. 2000). Interestingly, the interactions of zinc with the membrane do not induce alterations in membrane fluidity or lipid clustering. Using liposomes as a membrane model, we observed that zinc can act jointly with α-tocopherol (an antioxidant present in the hydrophobic portion of the membrane) and the water-soluble antioxidant epicatechin to inhibit iron-supported lipid oxidation (Zago and Oteiza, 2001).

### *Polyphenols can interact and reduce the access of oxidants and other deleterious molecules*

Polyphenols have a well-recognized antioxidant capacity both *in vitro* and *in vivo* (Rice-Evans 2001). This antioxidant activity has been attributed mainly to their capacity to scavenge oxygen and nitrogen active species (Bors et al. 1990) and to chelate redox-active metals (van Acker et al. 1998). The relevant chemical characteristics that contribute to the flavonoids' antioxidant/oxidants scavenging activity are: (a) the 3′,4′ hydroxyl (catechol) groups in the B ring, (b) the 2,3 double bond in conjugation with a 4-oxo group in the C ring, and (c) the presence of hydroxyl groups in positions 3 and 5 (Bors et al. 1990, Heim et al. 2002).

The interaction of polyphenols with bilayers could be a relevant mechanism in the protection by flavonoids of membrane oxidation. For flavonoids that partition in the non-polar region of the bilayer, their antioxidant activity can be attributed to both their capacity to interact with free radicals and, similarly to vitamin E, inhibit the propagation of lipid oxidation or, by increasing membrane fluidity.

The antioxidant activity of quercetin, hesperetin, naringenin and rutin has been reported to be related to their capacity to induce a decrease in $T_m$ in dipalmitoylphosphatidylcholine liposomes (Saija et al. 1995, Pawlikowska-Pawlega et al. 2003), indicative of a less ordered membrane. On the contrary, an increase in membrane lipid packing has been described for the flavonoids and isoflavonoids naringenin, rutin, genistein, genistin, biochanin A, equol, 4-hydroxy equol, dihydrodaidzein and dehydrogenistein in phosphatidylcholine liposome membranes (Arora et al. 2000). These opposite findings may be due to the different composition of the membranes under study. In the first study (Saija et al. 1995), the use of dipalmitoylphosphatidylcholine liposomes resulted in an ordered phase that was interrupted by the incorporation of the flavonoids between the acyl chains of the phospholipids. However, in the second study (Arora et al. 2000) 1-stearoyl-2-linoleoyl phosphatidylcholine liposomes were used, which by the geometry of the unsaturated fatty acid, results in a less ordered phase. In this case, the incorporation of the flavonoid in the bilayer core determined a higher ordering of the acyl chains, thus increasing the $T_m$ of the membrane. Due to the limited information on the effects of flavonoids on membrane lipid packing, further research is required to assess the influence of the different flavonoid families on the fluidity of biological membranes, and its potential role on lipid oxidation rates and membrane-associated events.

Given the above, we tested the hypothesis that certain flavonoids can interact with the lipid polar head groups at the lipid–water interface of membranes and protect the bilayer against the aggression of deleterious molecules. If the damaging molecule is an oxidant, this protective effect could contribute to the overall antioxidant action of certain flavonoids.

For this work, the experimental model was liposomes (composed of brain phosphatidylcholine and phosphatidylserine at a molar ratio 60:40 (PC/PS)) containing the fluorescent probe octadecyl rhodamine B chloride (Molecular Probes Incorporation, Eugene, OR, USA) at concentrations of self-quenching (Domecq et al. 2001). The addition of a detergent (Triton X-100) causes the progressive disruption of the membrane and the transition of a liposome to micelle structure. This transition is followed as the increase in fluorescence intensity due to the physical separation of the probe with the subsequent decrease in fluorescence auto-quenching. Liposomes were incubated for 2 min with different flavonoids (flavanols, flavonols, isoflavones, and flavanones), phenylpropanones and benzoic acid derivatives (Figure 1) and subsequently added with increasing amounts of Triton X-100. Figure 2 shows typical curves of the changes in fluorescence intensity as a function of the concentration of added detergent in the absence or the presence of kaempferol, naringenin, epigallocatechin, epigallocatechin gallate and genistein. From similar curves the $C_{50}$ value (concentration of Triton X-100 required to achieve 50% of the maximum fluorescence intensity) were calculated for different components of the flavonoid families, and for phenylpropanols and benzoic acid derivatives (Table I). From the tested compounds, the more hydrophilic flavonoids, flavonols and flavanols, had a significant protective effect on the detergent-mediated disruption of the bilayer. The formation of hydrogen bonds between the flavonoids and the polar head groups of the membrane lipids at the interface in part determines the capacity of these compounds to interact with the membrane surface and protect it from external damage. The number and distribution of the hydroxyl groups and the tridimensional structure of the flavonoids determine the formation of hydrogen bonds. Among the flavonols the membrane protective effects depend on the number

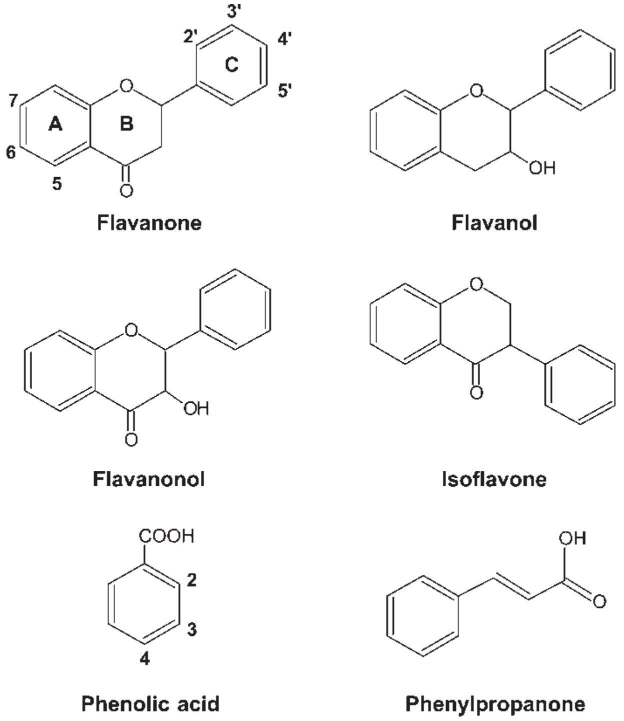

Figure 1. Chemical structure of the studied flavonoid families investigated.

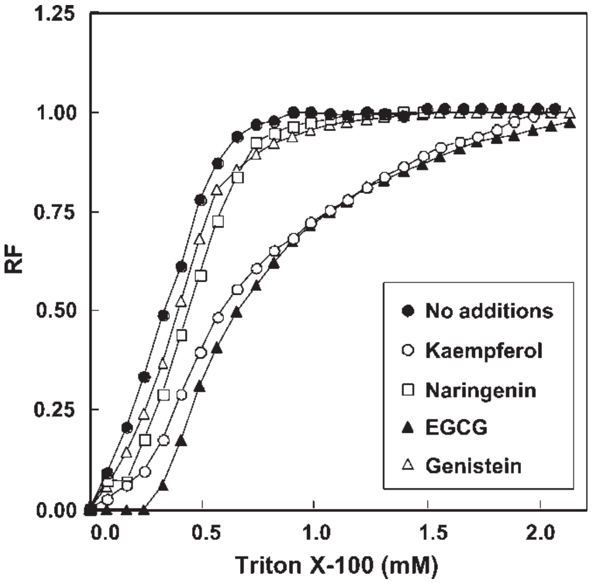

Figure 2. *Flavonoids reduce liposome disruption by Triton X-100.* PC/PS liposomes (60:40 molar ratio) containing 3 mol% of the fluorescent probe octadecyl rhodamine were incubated at 25°C for 5 min with or without the addition of 25 μM kaempferol, naringenin, epigallocatechin gallate (EGCG) or genistein. Increasing amounts of Triton X-100 were added every 2 min under continuous stirring and the fluorescence emission at 580 nm ($\lambda_{excitation}$: 560 nm) was registered. The addition of the detergent was continued until a constant fluorescence was reached, which corresponded to the micellar form of the lipids. The curves correspond to one representative experiment ($n = 4$).

Table I. Capacity of flavonoids to prevent membrane disruption by a detergent (Triton X-100)[*,†].

| Family | | Substitutions | | | | | | | | |
|---|---|---|---|---|---|---|---|---|---|---|
| | | 3 | 5 | 6 | 7 | 2′ | 3′ | 4′ | 5′ | $C_{50}$ |
| Flavanones | Flavanone | H | H | H | H | H | H | H | H | 0.310 ± 0.011 |
| | Naringenin | H | OH | H | OH | H | H | OH | H | 0.328 ± 0.019 |
| | Hesperetin | H | OH | H | OH | H | OH | OMe | H | 0.311 ± 0.015 |
| Flavanols | Catechin | OH | OH | H | OH | H | OH | OH | H | 0.330 ± 0.011 |
| | Epicatechin | OH | OH | H | OH | H | OH | OH | H | 0.323 ± 0.012 |
| | Epigallocatechin | OH | OH | H | OH | H | OH | OH | OH | 0.350 ± 0.014[a] |
| | Epigallocatechin gallate | GA | OH | H | OH | H | OH | OH | OH | 0.626 ± 0.020[b] |
| Flavonols | Kaempferol | OH | OH | H | OH | H | H | OH | H | 0.661 ± 0.024[b] |
| | Morin | OH | OH | H | OH | OH | H | OH | H | 0.715 ± 0.019[c] |
| | Galangin | OH | OH | H | OH | H | H | H | H | 0.473 ± 0.026[d] |
| Flavanonols | Taxifolin | OH | OH | H | OH | H | OH | OH | H | 0.308 ± 0.004 |
| Isoflavones | Daidzein | | H | H | OH | H | H | OH | H | 0.293 ± 0.008 |
| | Genistein | | OH | H | OH | H | H | OH | H | 0.338 ± 0.007 |
| | | | 2 | | 3 | | 4 | | | |
| Phenolic acids | Vanillic acid | | H | | OMe | | OH | | | 0.324 ± 0.011 |
| | Protocatechuic acid | | H | | OH | | OH | | | 0.303 ± 0.008 |
| Phenyl propanones | Cinnamic acid | | H | | H | | H | | | 0.305 ± 0.009 |
| | *p*-Coumaric acid | | H | | H | | OH | | | 0.307 ± 0.015 |
| No additions | | | | | | | | | | 0.307 ± 0.008 |

[*] Values represent means ± SEM, $n = 4$, values having superscripts are significantly different from No additions (one-way ANOVA test, $p < 0.05$). Different superscripts indicate that values are significantly different among them.
[†] Experiments were carried out in PC/PS liposomes as described in the legend to Fig. 2. The final concentration of all flavonoids was 25 μmol/l. $C_{50}$ was calculated as the amount of Triton X-100 required to attain the half of the maximum fluorescence intensity.

of hydroxyl groups in the molecule. Thus, the $C_{50}$ value for kaempferol, morin and galangin correlated positively ($r$: 0.89, $p < 0.001$) with the number of hydroxyl groups in the molecule. At the same number of hydroxyl groups, catechin, epicatechin and taxifolin had no effect, while morin had the highest protective effect of all the flavonoids tested. This indicates that

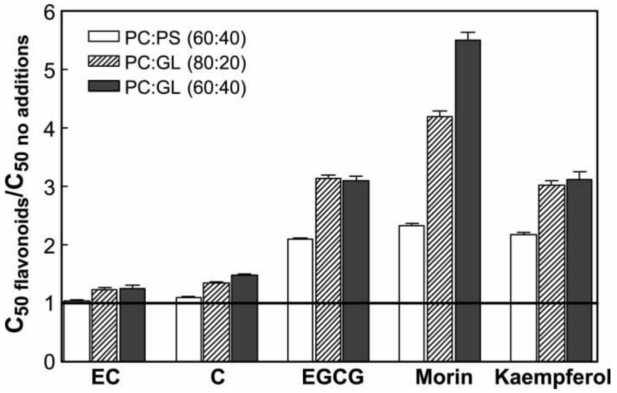

Figure 3. *Galactolipids–flavonoids interactions enhance the capacity of flavonoids to prevent liposome disruption by Triton X-100.* PC/PS (60:40), PC/galactolipids (GL) (80:20), or PC/GL (60:40) liposomes containing 3 mol% of the fluorescent probe octadecyl rhodamine were incubated at 25°C for 5 min with or without the addition of 25 μM epicatechin (EC), catechin (C), EGCG, morin or kaempferol. After incubation, liposomes were progressively disrupted by the addition of Triton X-100 as described in the legend to Fig. 1. Results are expressed as the ratio between the $C_{50}$ obtained in the presence and in the absence of the assessed flavonoids, and are the mean ± SEM of four independent experiments.

while the methoxy group at position 4 of the C ring does not contribute to the capacity of these compounds to form hydrogen bonds, the presence of the 2,3 double bond at the C ring is a fundamental contributing factor. Among the flavanols, the presence of an additional hydroxyl group in epigallocatechin determines a significant increase in membrane protection, and a gallate residue in position 3 (epigallocatechin gallate), which adds three hydroxyl groups to the molecule, is associated with a 96% increase in $C_{50}$ compared to epicatechin (Table I). In agreement with these results, the glycosylated derivatives (galactoside, glucoside, lactoside and maltoside) of the flavonolignan silybin showed higher protective effects toward tert-butylhydroperoxide-induced lipid oxidation in rat liver mitochondria, compared to the parent compound silybin (Kosina et al. 2002). Significantly, the glycosides were weaker electron donors that sylibin per se.

The nature of the interactions established between flavonoids and the lipid bilayer that prevented liposome disruption by Triton X-100 was also investigated. For that purpose, liposomes composed of PC and a natural polyhydroxylated lipid (galactolipids, GL) in a 80:20 or 60:40 molar ratio, and containing the fluorescent probe octadecyl rhodamine were incubated in the presence of those flavonoids that displayed the highest protection in PC/PS (60:40) liposomes. GL caused only a mild increase in catechin- and epicatechin-mediated $C_{50}$ displacement (18 and 21% increase for epicatechin, and 23 and 34% for catechin at 20 and 40 mol% GL, respectively) compared to PC/PS (60:40) liposomes

(Figure 3). The presence of a gallate group in epigallocatechin gallate substantially increased $C_{50}$ at 20 mol% GL (50%), while at 40% GL no additional increase was observed (Figure 3). Among the flavonols, kaempferol caused a 40% increase in $C_{50}$ at 20% GL, effect that was not further modified by increasing GL concentration. Morin had the highest effect on membrane protection, causing a 80 and 136% increase in $C_{50}$ at 20 and 40% GL, respectively (Figure 3). Thus, the protective effect of these flavonoids against Triton X-100-mediated liposome disruption was higher in the GL-containing membranes respect to PC/PS (60:40) liposomes (Figure 3), effect that increased with the amount of GL in the membrane. In addition, the magnitude of flavonoids effects strongly depended on the number of hydroxyl groups present in each moiety. These findings support the involvement of hydrogen bonding between flavonoids and the polar headgroup of lipids at the water–lipid interface of membranes.

Several lines of evidence suggest a protective effect of flavonoids in biological systems. It has been observed that the capacity of certain flavonoids to prevent oxidant-induced permeabilization of rat liver lysosomes is not always related to their inhibitory action on lipid oxidation (Decharneux et al. 1992). Some of the tested flavonoids could prevent the leakage of *n*-acetyl glucosaminidase in lysosomes incubated in isosmotic glucose (Decharneux et al. 1992). In agreement with results shown in Table I, while kaempferol and morin were strong inhibitors, naringenin and taxifolin had minor effects against osmotic stress. Similar results were obtained for diosmentin, the main metabolite of the flavonoid diosmin (Villa et al. 1992). Diosmentin inhibited the release of the enzymes lactate dehydrogenase and aspartate-aminotransferase from primary hepatocyte cell cultures induced by non-oxidant (erythromycin estolate) and pro-oxidant (tert-butylhydroperoxide) aggressors (Villa et al. 1992). A direct protective action of flavonoids *in vivo* is supported by the observation that erythrocytes obtained from rats given a flavanoid-rich meal (cocoa extract) showed a reduced susceptibility to free radical-mediated hemolysis (Zhu et al. 2002).

Contrasting to the protective effects of flavonoids on membranes, a bactericidal action of epigallocatechin gallate was attributed to its capacity to affect membrane permeability (Ikigai et al. 1993). This and others reports stress the necessity of further research to understand the role of flavonoid-membrane interactions on the biological effects of these compounds.

### Conclusions

Flavonoids and related polymers can have different types of interactions with membranes depending on their chemical structure. The more hydrophobic flavonoids can partition in the hydrophobic core of the membranes. Some of the possible consequences of this interaction are a direct modulation of membrane physical properties on the capacity of flavonoids to efficiently scavenge free radicals, in part inhibiting the lipid oxidation chain reaction. The more hydrophilic flavonoids interact by hydrogen bonding with the polar head groups at the lipid–water interface of membranes. This type of interaction may provide a level of protection for the bilayer from external and internal aggressors (i.e. oxidants) contributing to preserve the structure and function of biological membranes.

### Acknowledgements

This work was supported by grants from the University of Buenos Aires (B054 and B042), and CONICET (PIP 02120 and 0738/98) Argentina and from Mars Incorporated (Hackettstown, NJ). A.G. Erlejman is a graduate fellow from the University of Buenos Aires, Argentina.

### References

Arora A, Byrem TM, Nair MG, Strasburg GM. 2000. Modulation of liposomal membrane fluidity by flavonoids and isoflavonoids. Arch Biochem Biophys 373:102–109.

Bors W, Heller W, Michel C, Saran M. 1990. Flavonoids as antioxidants: Determination of radical-scavenging efficiencies. Methods Enzymol 186:343–355.

Bravo L. 1998. Polyphenols: Chemistry, dietary sources, metabolism and nutritional significance. Nutr. Rev 56:317–333.

Castelli F, Uccella N, Trombetta D, Saija A. 1999. Differences between coumaric and cinnamic acids in membrane permeation as evidenced by time-dependent calorimetry. J Agric Food Chem 47:991–995.

Cervato G, Viani P, Masserini M, Di Iorio C, Cestaro B. 1988. Studies on peroxidation of arachidonic acid in different liposomes below and above phase transition temperature. Chem Phys Lipids 49:135–139.

Decharneux T, Dubois F, Beauloye C, Wattiaux-De Coninck S, Wattiaux R. 1992. Effect of various flavonoids on lysosomes subjected to an oxidative or an osmotic stress. Biochem Pharmacol 44:1243–1248.

van Dijk C, Driessen AJ, Recourt K. 2000. The uncoupling efficiency and affinity of flavonoids for vesicles. Biochem Pharmacol 60:1593–1600.

Domecq A, Disalvo EA, Bernik DL, Florenzano F, Politi MJ. 2001. A stability test of liposome preparations using steady-state fluorescent measurements. Drug Deliv 8:155–160.

Exon JH, South EH. 2003. Effects of sphingomyelin on aberrant colonic crypt foci development, colon crypt cell proliferation and immune function in an aging rat tumor model. Food Chem Toxicol 41:471–476.

Hashimoto M, Hossain MS, Shimada T, Yamasaki H, Fujii Y, Shido O. 2001. Effects of docosahexaenoic acid on annular lipid fluidity of the rat bile canalicular plasma membrane. J Lipid Res 42:1160–1168.

Heim KE, Tagliaferro AR, Bobilya DJ. 2002. Flavonoid antioxidants: Chemistry, metabolism and structures-activity relationships. J Nutr Biochem 13:572–584.

Hendricks JJA, de Vries HE, van der Pol SMA, van der Berg TK, van Tol EAF, Dijkstra CD. 2003. Flavonoids inhibit myelin phagocytosis by macrophages; a structure-activity relationship study. Biochem Pharmacol 65:877–885.

Kosina P, Kren V, Gebhardt R, Grambal F, Ulrichova J, Walterova D. 2002. Antioxidant properties of silybin glycosides. Phytother Res 16:S33–S39.

Kuo P, Weinfeld M, Loscalzo J. 1990. Effect of membrane fatty acyl composition on LDL metabolism in Hep G2 hepatocytes. Biochemistry 29:6626–6632.

Lim BO. 2003. Effects of wogonin, wogonoside, and 3,5,7,2′,6′-pentahydroxyflavone on chemical mediator production in peritoneal exudates cells and immunoglobulin E of rat mesenteric lymph node lymphocytes. J Ethnopharmacol 84:23–29.

Lin N, Sato T, Takayama Y, Minaki Y, Sashida Y, Yano M, Ito A. 2003. Novel anti-inflammatory actions of nobiletin, a citrus polymethoxy flavonoid, on human synovial fibroblasts and mouse macrophages. Biochem Pharmacol 65:2065–2071.

Lotito SB, Fraga CG. 1998. (+)-Catechin prevents human plasma oxidation. Free Radic Biol Med 24:435–441.

Lotito SB, Actis-Goretta L, Renart ML, Caligiuri M, Rein D, Schmitz HH, Steinberg FM, Keen CL, Fraga CG. 2000. Influence of oligomer chain length on the antioxidant activity of procyanidins. Biochem Biophys Res Commun 276:945–951.

McLean LR, Hagaman KA. 1992. Effect of lipid physical state on the rate of peroxidation of liposomes. Free Radic Biol Med 12:113–119.

Movileanu L, Neagoe I, Flonta ML. 2000. Interaction of the antioxidant flavonoid quercetin with planar lipid bilayers. Int J Pharm 205:135–146.

Mowri H, Nojima S, Inoue K. 1984. Effect of lipid composition of liposomes on their sensitivity to peroxidation. J Biochem (Tokyo) 95:551–558.

Niranjan TG, Krishnakantha TP. 2000. Membrane changes in rat erythrocyte ghosts on ghee feeding. Mol Cell Biochem 204:57–63.

Ollila F, Halling K, Vuorela P, Vuorela H, Slotte JP. 2002. Characterization of flavonoid–biomembrane interactions. Arch Biochem Biophys 399:103–108.

Oteiza PI. 1994. A mechanism for the stimulatory effect of aluminum on iron-induced lipid peroxidation. Arch Biochem Biophys 308:374–379.

Pawlikowska-Pawlega B, Gruszeckib WI, Misiakb LE, Gawron A. 2003. The study of the quercetin action on human erythrocyte membranes. Biochem Pharmacol 66:605–612.

Ratty AK, Sunamoto J, Das NP. 1988. Interaction of flavonoids with 1,1-diphenyl-2-picrylhydrazyl free radical, liposomal membranes and soybean lipoxygenase-1. Biochem Pharmacol 37:989–995.

Rice-Evans C. 2001. Flavonoid antioxidants. Curr Med Chem 8:797–807.

Saija A, Scalese M, Lanza M, Marzullo D, Bonina F, Castelli F. 1995. Flavonoids as antioxidant agents: Importance of their interaction with biomembranes. Free Radic Biol Med 19:481–486.

Terao J, Piskula M, Yao Q. 1994. Protective effect of epicatechin, epicatechin gallate, and quercetin on lipid peroxidation in phospholipid bilayers. Arch Biochem Biophys 308:278–284.

Tomassoni ML, Amori D, Magni MV. 1999. Changes of nuclear membrane lipid composition affect RNA nucleocyto-plasmic transport. Biochem Biophys Res Commun 258:476–481.

Tonks A, Morris RHK, Price AJ, Thomas AW, Jones KP, Jackson SK. 2001. Dipalmitoylphosphatidylcholine modulates inflam-matory functions of monocytic cells independent of mitogen activated protein kinases. Clin Exp Immunol 124:86–94.

Tsuchiya H. 2001. Stereospecificity in membrane effects of catechins. Chem Biol Int 134:41–54.

van Acker SA, van Balen GP, van den Berg DJ, Bast A, van der Vijgh WJ. 1998. Influence of iron chelation on the antioxidant activity of flavonoids. Biochem Pharmacol 56:935–943.

Verstraeten SV, Oteiza PI. 2000. Effects of $Al^{3+}$ and related metals on membrane phase state and hydration: Correlation with lipid oxidation. Arch Biochem Biophys 375:340–346.

Verstraeten SV, Golub MS, Keen CL, Oteiza PI. 1997a. Myelin is a preferential target of aluminum-mediated oxidative damage. Arch Biochem Biophys 344:289–294.

Verstraeten SV, Nogueira LV, Schreier S, Oteiza PI. 1997b. Effect of trivalent metal ions on phase separation and membrane lipid packing: Role in lipid peroxidation. Arch Biochem Biophys 338:121–127.

Verstraeten SV, Keen CL, Golub MS, Oteiza PI. 1998. Membrane composition can influence the rate of $Al^{3+}$-mediated lipid oxidation: effect of galactolipids. Biochem J 333:833–838.

Verstraeten SV, Keen CL, Schmitz HH, Fraga CG, Oteiza PI. 2003. Flavan-3-ols and procyanidins protect liposomes against lipid oxidation and disruption of the bilayer structure. Free Radic Biol Med 34:84–92.

Villa P, Cova D, De Francesco L, Guaitani A, Palladini G, Perego R. 1992. Protective effect of diosmetin on *in vitro* cell membrane damage and oxidative stress in cultured rat hepatocytes. Toxicology 73:179–189.

Zago MP, Oteiza PI. 2001. The antioxidant properties of zinc: Interactions with iron and antioxidants. Free Radic Biol Med 31:266–274.

Zago MP, Verstraeten SV, Oteiza PI. 2000. Zinc in the prevention of $Fe^{2+}$-initiated lipid and protein oxidation. Biol Res 33:143–150.

Zhu QY, Lazarus SA, Holt RR, Orozco TJ, Keen CL. 2002. Inhibitory effects of cocoa flavonoids on free radical-induced erythrocyte hemolysis. Exp Biol Med 227:321–329.

